# Current Concepts in Community and Ventilator Associated Lower Respiratory Tract Infections in ICU Patients

**DOI:** 10.3390/antibiotics9070380

**Published:** 2020-07-05

**Authors:** Ignacio Martin-Loeches

**Affiliations:** 1 Department of Anaesthesia and Critical Care Medicine, St James’s Hospital, D08 X4RX Dublin, Ireland; drmartinloeches@gmail.com; 2 Multidisciplinary Intensive Care Research Organization (MICRO), St James’s Hospital, D08 X4RX Dublin, Ireland; 3 Pulmonary Intensive Care Unit, Respiratory Institute, Hospital Clinic of Barcelona, IDIBAPS, University of Barcelona, CIBERes, 08036 Barcelona, Spain

**Keywords:** sepsis, pneumonia, VA-LRTI, VAP

## Abstract

It is widely known that pneumonia (either community acquired or hospital acquired, as like ventilator associated pneumonia (VAP)), is the most frequent type of severe infection and continues to pose a significant burden on healthcare services worldwide. Despite new diagnostic developments, most pneumonia cases continue to be difficult to diagnose clinically, partly due to acquired antibiotic resistance and the lack of a ‘gold standard’ method of diagnosis. In other words, the lack of a rapid, accurate diagnostic test, as well as the uncertainty of the initial etiologic diagnosis and the risk stratification, results in empirical antibiotic treatments. There are significant changes in the aetiology of patients with ventilator associated lower respiratory tract infections (VA-LRTI), which are characterised by a higher incidence of multi drug resistant organisms. Evidence suggests that when patients with VA-LRTI develop organ failure, the associated mortality can be exceptionally high with frequent complications, including acute respiratory distress syndrome, acute kidney injury, and septic shock. Appropriate antibiotic treatments must consider that the present cardiovascular failure seen in patients has a different association with the patient’s mortality. Unlike patients with less severe clinical presentations, who have a higher chance of survival when the appropriate antibiotics are administered promptly, for patients with a severe subtype of the disease, the appropriateness of antibiotic treatment will impact the patient’s outcome to a lesser extent. The present review highlights certain factors detectable at the time of admission that could indicate patients who are at a high risk of bacteraemia and who, therefore, merit more intense therapy and stratified care.

## 1. Introduction

The most frequent medical admissions to intensive care units (ICU) result from the development of respiratory failure, mainly due to infections that are either community acquired or have a nosocomial origin [1]. Patients with community acquired pneumonia admitted to the ICU who require either invasive mechanical ventilation or vasopressors present high mortality rates. Current guidelines for community and ventilator-associated respiratory tract infections (VA-LRTI) emphasize the importance of prompt broad-spectrum antimicrobial therapy and a combination approach (due to the high severity of the disease) to target the most frequent pathogens. However, in recent years, we have observed a surge of “non-common” pathogens, such as Gram-negative bacilli [2]. There is an inherent risk with this strategy that the liberal use of antimicrobial combinations will encourage the emergence of polymicrobial resistant organisms and generate untreatable infections. Given the recent significant fall-off in the discovery of next-generation novel anti-bacterial agents by the pharmaceutical industry, the emergence of antibiotic-resistant pathogens has led global leaders to warn of a future where common bacterial infections become untreatable and oftentimes fatal. This is becoming especially worrisome in non-Western countries.

The clinical course of ICU patients may be complicated by a large spectrum of lower respiratory tract infections (LRTI), defined by different clinical and microbiological aspects [3]. Interestingly, patients admitted to an ICU that require invasive mechanical ventilation after developing pneumonia in the ward (ventilated Hospital acquired pneumonia) are on the rise [4]. Concepts such as organ failure in patients with VA-LRTI are being incorporated into guidelines as a risk factor for the acquisition of multidrug resistant organisms (MDRO). In the present manuscript, current information on both community acquired and nosocomial pneumonia is provided, with a focus on the risk factors that can stratify patients, the use of biomarkers, the benefit of pharmacokinetic-guided antibiotic administration, and how clinical scoring determines whether patients are admitted to the ICU. 

## 2. Changes in Epidemiology in Community Acquired Pneumonia

Worldwide, *Streptococcus pneumoniae* continues to be the most frequent causative pathogen in community acquired pneumonia (CAP), independent of the age of patients, comorbid conditions, or clinical settings (namely, outpatients vs. hospitalised patients). However, in ICU settings, it is becoming more clear that there are more mixed aetiologies and fewer atypical pathogens [5]. In recent years, it has been observed that the aetiology of CAP is related to resistant pathogens, independent of whether they are Enterobacteriaceae or not. For this reason, information pertaining to PES (*Pseudomonas aeruginosa*, extended-spectrum β-lactamase *Enterobacteriaceae*, and methicillin-resistant *Staphylococcus aureus* (MRSA)) pathogens has been recently published [6]. Notably, P. aeruginosa and MRSA are the most frequently reported pathogens and require antibiotic therapies that differ from those recommended by the majority of guidelines. There are some factors that hint at the risk of PES infection: an age >65 years, previous antibiotic use, chronic conditions (a chronic respiratory disorder or chronic renal disease), and unconsciousness. These risk factors have been computed into a score by Prina et al. [7], with an area under the curve for the score of 0.759 (a 95% confidence interval, 0.713–0.806; *p* < 0.001). In addition, PES pathogens were independently associated with an increased risk of 30-day mortality (odds ratio = 2.51).

Patients with CAP often develop mild forms of the disease, and only a minority are hospitalised. When this occurs, there is a small subgroup of patients that require intensive care management, typically due to septic shock or the need to provide invasive mechanical ventilation. Villafuerte et al. [8] analysed the data of the 3193 patients enrolled in the Global initiative for methicillin-resistant *Staphylococcus aureus* pneumonia (GLIMP) study with CAP to determine the prevalence of Enterobacteriaceae and refine the classification of the genera and species within this family in infected patients. The authors found that only 6% of the patients developed a CAP due to Enterobacteriaceae, with half of them resistant to at least one antibiotic class. The data identified several risk factors important to Enterobacteriaceae acquisition: severe CAP, being underweight, and prior extended-spectrum beta-lactamase (ESBL) infection. 

Viruses also play an important role in CAP patients. Zhou et al. [9] found that in 40% of patients admitted to 34 hospitals in mainland China, respiratory viruses could be detected by molecular methods. The influenza virus was detected in one third of the cases, with other respiratory viruses presenting at a much lower rate (respiratory syncytial virus (3.6%), adenovirus (3.3%), human coronavirus (3.0%), parainfluenza virus (2.2%), human rhinovirus (1.8%), and human metapneumovirus (1.5%)). When analysing patients being admitted to the ICU, Choi et al. [10] found that respiratory viruses caused severe CAP in 36.4% of the cases, but rhinovirus was the most commonly identified virus (23.6%), followed by the parainfluenza virus (20.8%), human metapneumovirus (18.1%), influenza virus (16.7%), and respiratory syncytial virus (13.9%). Hence, acquiring information about the type of infections, causative pathogens, and outcomes could aid in developing further preventive policies, diagnostic accuracy, treatment, and resource allocation.

## 3. Bacteraemic Episodes for Gram Negatives in CAP

In Western countries, CAP due to Gram-negative bacilli (GNB) is relatively rare. Bacteraemic episodes in patients with CAP are more frequent in patients who present severe forms of CAP, especially those admitted to an ICU. The presence of bacteraemia has also been associated with higher mortality rates. The main aetiology of bacteraemic episodes is bacteraemic pneumococcal pneumonia. Recent evidence has established that the presence of Enterobacteriaceae is uncommon in these GNB episodes. However, some studies of interest should be noted. Lin et al. [11] reported that K. pneumoniae were the dominant cause of bacteraemic CAP in Taiwanese hospitalised adults during the period of 2001–2008 and highlighted that septic shock and respiratory failure were independent risk factors for both early and total mortality in these patients. The authors reported that compared to pneumococcal bacteraemic, the episodes had a worse prognosis in those affected with *K. pneumoniae* CAP. Interestingly, K1 and K2 were the most frequent serotypes, but there were no significant differences due to K1/K2 and non-K1/K2 isolates in the clinical characteristics of patients with bacteraemic CAP, and the mortality was over 50%. Also Asia, Inghammar et al. [12] reported an 8.6 per cent prevalence of GNBs in all CAP patients with 30% bacteraemic episodes. The most frequent GNB identified was K. pneumoniae (30%), followed by *Burkholderia pseudomallei* (26%), *Pseudomonas aeruginosa* (19%), and *Escherichia coli* (9%). In patients with severe CAP, one out of ten cases were caused by GNBs, and there was a significantly higher mortality in patients with CAP due to GNB. 

## 4. Epidemiology in Other Rarely Reported Countries

In 2016, total of over 700,000 deaths were estimated by the World Health Organization (WHO) in Asia due to lower respiratory tract infections [13]. Unfortunately, the majority of granular data were published more than a decade ago (2002 and 2004) on aetiology, clinical outcomes, and risk factors by the Asian Network for Surveillance of Resistant Pathogens from a representative sample of Asian countries [14]. Two thirds of patients were hospitalized, and 10% were admitted to the ICU. In patients with Pneumonia Severity Index (PSI) categories 4 and 5, mortality ranged from 7.3% to 50.6%. *S. pneumoniae* was the pathogen most often isolated (29.2%), followed by atypical pathogens in 25% and Gram-negative bacteria (e.g., *K. pneumoniae* and *P. aeruginosa*) in 22%.

In addition, the 2016 Global Burden of Disease study [15] highlighted the substantial improvement in decreasing the burden of lower respiratory tract infections. However, there continues to be a knowledge gap in some countries, especially those in Sub-Saharan Africa, Southeast Asia, and South Asia. In these regions, mortality rates remain high. Here, S. pneumoniae is of lesser importance compared to western countries; GNB occurred in 13% of hospitalised CAP cases, being more frequent in India and Southeast Asia. Another pathogen of high relevance is *Mycobacterium tuberculosis* in Southeast Asia. In northeast Thailand, Burkholderia Pseudomallei was reported to be a major pathogen [16].

An important concept in some Asian regions is the overlap of fever syndromes and acute respiratory failure that leads to acute respiratory distress syndrome (ARDS). Malaria, Dengue, leptospirosis, and typhus should be included in the differential diagnosis. An important cause of severe CAP is B. pseudomallei (melioidosis), which is the second most common isolated pathogen in CAP patients admitted to hospitals in Thailand [17].

## 5. Impact of Aging in Community Acquired Pneumonia

Those aged over 65 years are defined as elderly, who experience a greater impact from pneumonia than younger age groups. The annual incidence of pneumonia among the elderly is four-times that of younger populations [18]. As in other age populations, the most common pathogen isolated in patients over 65 years with CAP is *Streptococcus pneumoniae*, although atypical and Gram-negative bacilli also play a major role [19]. Aspiration pneumonia is also a concern for the elderly. Published studies have found that age is an independent risk factor for the aspiration pneumonia that commonly occurs with dysphagia (neurovascular and medication induced, such as antipsychotics) [20]. Likewise, Cilloniz et al. [21] conducted a retrospective observational study and found that among patients with CAP, chronic renal and neurological diseases are both independent risk factors for mortality. Additionally, for patients more than 80 years of age who are hospitalised with CAP, their mortality rates increase if they develop sepsis. Likewise, Martin-Loeches et al. [22] conducted a prospective, observational, multicenter cohort study to determine the risk factors for mortality in elderly and very elderly critically ill patients with sepsis. The main finding was that age was an independent risk factor only in the group of patients over 80 years old. While these data are important, the focus of the paper was on patients with sepsis in general and not only those with CAP. 

## 6. Bacteraemic Episodes in Hospital Acquired Pneumonia 

Bacteraemic episodes can be frequent in patients with CAP. However, they are less common in pneumonia with a nosocomial origin. Bacteraemia has not been widely studied as a risk factor in patients with nosocomial pneumonia. Some studies show interesting features that can help us understand the epidemiology of bacteraemia in patients who are mechanically ventilated. Harmon et al. [23] reported a 11% incidence of bacteraemia in patients mechanically ventilated after a cardiac arrest in a targeted temperature management (TTM) trial. Gram-negative [*Klebsiella pneumoniae* (12.1%)] and Gram-positive pathogens [*Staphylococcus aureus* (24.2%)] were both prevalent. Agbaht et al. [24] performed a retrospective, single-centre, observational cohort study that assessed the association of mortality in patients with bacteraemia. In the design of the study, the authors matched bacteraemic patients presenting ventilator-associated pneumonia (VAP) with two controls using VAP and negative blood cultures. The authors estimated that the relative risk of death for bacteraemic cases was 2.86%, since mortality for cases and matched NB-VAP controls was 40.6% (13 of 32) and 19.3% (11 of 57), respectively. More recently, Magret et al. [25] conducted a secondary analysis of a large multicenter cohort of patients with HAP. From 689 patients with nosocomial pneumonia (465 VAP and 224 HAP), 15% of patients presented a bacteraemic episode. They also found that the mortality was two times (57.1%) that of patients without bacteraemia (33%). In addition, infection-related factors, such as aetiology, and predisposition factors, such as diagnostic category at admission, were associated with a higher risk of bacteraemia. Interestingly, *Acinetobacter baumannii*’s aetiology was associated with bacteraemic episodes.

## 7. Multidrug Resistant Organism Epidemiology in Nosocomial Pneumonia

Gram negative infections currently account for the majority of infection episodes. Vincent et al. [1] recently published the prevalence and outcomes of infection among patients in Intensive Care Units. This was an observational 24-hour point prevalence study conducted over 1150 centres in 88 countries. The authors reported that Gram-negative microorganisms were identified in 67% of the patients with infection. One of the most important findings was that MDRO were common. Moreover, *Klebsiella* sp. resistant to β-lactam antibiotics, including third-generation cephalosporins and carbapenems or carbapenem-resistant *Acinetobacter species*, were independently associated with a higher risk of death when compared to infection with another microorganism. 

One of the most important strategies in current medical practice is to better identify patients at risk of acquiring MDRO. There are some well-known risk factors, but the use of scores might help identify the most common MDROs: third-generation cephalosporin-resistant *Enterobacteriaceae* (3GC-R), carbapenem-resistant *Enterobacteriaceae* (CRE), and multidrug-resistant *Pseudomonas aeruginosa* (MDRP). Lodise et al. [26] recently published a clinical bedside tool that was used to retrospectively analyse a large database (almost 125,000 patients), including hospitalised patients with complicated urinary tract infections (cUTIs), complicated intra-abdominal infections (cIAIs), HAP/VAP, or bloodstream infections (BSIs) due to Gram-negative bacteria. The authors found that the most important predictors of an 3GC-R, CRE, or MDRP infection were previous administrations of antibiotics, the infection site, the presence of infection within the previous 3 months, and the hospital’s prevalence of 3GC-R, CRE, or MDRP. Martin-Loeches et al. analysed the probability of MDROs in patients with HAP [27]. Interestingly, in previous years, the classification of HAP was split into two categories. Patients with early onset (Eo) and late onset (Lo) HAP. The main difference was the time until pneumonia development and if the patient had risk factors for MDRO. This study found that there were no significant differences in the presence of potential resistant microorganisms (PRM) in Eo vs. Lo HAP episodes. Moreover, the major determinants of PRM were the occurrence of septic shock and the prevalence of more than 25% PRM for HAP episodes in the participating ICU. These findings have been included in clinical guidelines. Currently, there are two major clinical guidelines for HAP: American [28] and European [29]. While both guidelines have some differences, they also offer some common points regarding the aetiology and severity of the disease in patients with nosocomial pneumonia. 

An important element in controlling the spread of multidrug resistant organisms in hospital settings is the adoption of antibiotic stewardship programs (ASP). The main principles of ASP are to promote the appropriate use of antimicrobials (including antibiotics), improve patient outcomes, reduce antimicrobial resistance, and decrease the spread of infections caused by multidrug-resistant organisms. There are many ways to implement such programs, but a multidisciplinary approach with coordination among the attending physicians, nurses, and pharmacists is the most important factor [30,31]. In the following section, we further discuss the roles of the biomarkers that are a key element of ASP. 

## 8. The Role of Biomarkers in Ventilator Associated Lower Respiratory Tract Infections

Biomarkers play a major role in the diagnosis and management of severe infections in the ICU. Ventilator-associated lower respiratory tract infections (VA-LRTI) consist of ventilator-associated pneumonia (VAP) and ventilator-associated tracheobronchitis (VAT). Coelho et al. [32] analysed over 400 patients with VA-LRTI to distinguish the inflammatory responses of patients with either VAP or VAT. The authors found that procalcitonin (PCT) and C-reactive protein (CRP) presented lower values in patients with VAT compared to those with VAP; however, this difference was not significant. Both biomarkers could not help distinguish between VAT and VAP, as there was a marked overlap between the two. Regarding, VAP resolution, Povoa et al. [33] aimed to analyse the kinetic performance of several biomarkers (C-reactive protein (CRP), procalcitonin (PCT), and the mid-region fragment of pro-adrenomedullin (MR-proADM)) in a prospective, multicenter, observational study of microbiologically documented VAP. The biomarker that demonstrated added value in diagnostic precision was CRP. CRP kinetics were found to be useful as early as four days after the initiation of treatment to identify VAP patients with poor outcomes. On the other hand, Giamarellos et al. [34] developed a prognostic score of bacteraemia using the resolution of carbapenem-resistant Gram-negative bacteria (CRGNB) with a test and validation cohort. The authors described three clinical scenarios: (a) On day 2, Procalcitonin (PCT) decreased by more than 30%, and PCT on day 4 was below 0.5 ng/mL; (b) PCT on day 4 decreased by more than 40%, and PCT on day 4 was below 0.5 ng/mL; and (c) PCT on day 2 decreased by more than 30%, and PCT on day 4 decreased by more than 40%. However, the absence of these biomarkers is underpinned by our fundamental ignorance of patients’ immune and inflammatory responses in this area of medicine.

## 9. Vulnerable Patients at Risk for Nosocomial Infections

Patients with a special incidence of Gram-negative infections had comorbid conditions. Among conditions, cancer is one of the most challenging. The number of cancer patients admitted to ICUs is expected to increase due to several factors, such as aging and recent advances in therapy that have prolonged life expectancy. There are few studies that focus on patients with cancer. Notably, Stoclin et al. [35] recently reported a VAP rate of 24.5/1000 ventilator days among more than 3000 patients. This is clearly far higher than the results of studies conducted on non-cancer patients [36]. Interestingly, GNBs were the most common pathogens and occurred in almost half of the VAP episodes. Similarly, Moreau et al. [37] analysed the incidence of VA-LRTI in immunocompromised patients compared with non-immunocompromised patients. The authors found a lower incidence of VA-LRTI in immunocompromised patients for both VAP and VAT. The study also found that the rates of MDRO (72% versus 59%; *p* = 0.011) and mortality while in the ICU (54% versus 30%; *p* < 0.0001) were higher for immunosuppressed patients compared to those who were not immunosuppressed. This result has two consequences: On the one hand, MDRO have a higher risk of developing VA-LRTI in more vulnerable hosts, and, when this happens, the outcome is worse. 

The data produced in the last decade are interesting for patients with other comorbid conditions, such as chronic obstructive pulmonary disease (COPD). Koulenti et al. [38] found COPD among 20% of European ICU admissions. The authors not only reported a higher incidence of mortality compared to non COPD patients but also a much longer ICU stay. Regarding aetiology, it is unsurprising that Pseudomonas aeruginosa (PA) was more common in COPD patients with VAP. However, what is clinically useful is that PA occurred in Early onset-VAP (Eo-VAP) 2.5 times more frequently than in patients without early-onset VAP. On the other hand, a cohort study by Rouze et al. [39] found that among COPD patients mechanically ventilated in Europe and Latin America, neither VAP nor VAT were more frequent. Moreover, among patients with VA-LRTI, Escherichia coli and Stenotrophomonas maltophilia were found at significantly higher rates in COPD patients. The authors did not observe worse outcomes for patients with COPD. These are among the largest multicentre studies performed on patients with COPD, and the results seems to be contradictory. The selected population and the exclusion of trauma patients in the paper by Koulenti et al. may partially explain these differences. 

## 10. Organ Failure Severity in Nosocomial Pneumonia

Organ failure is an important determinant of severity in patients with nosocomial pneumonia. Among organ failure, there are three types of major interest: Acute kidney injury (AKI), ARDS, and cardiovascular failure. Younan et al. [40] analysed trauma patients admitted to an academic U.S. hospital. Almost 1500 patients were admitted over the two years of the study period and reported a low incidence of VAP at 3%. However, one of the most interesting findings was that patients with VAP presented a higher incidence of AKI and longer ICU stays than those without VAP. Whether the VAP per se or the development of AKI determined these longer ICU stays remains a question.

One main concern in patients with VA-LRTI is the development of ARDS during their ICU stay. Zampieri et al. [41] reported a 17% incidence of ARDS in patients who received invasive mechanical ventilation (MV) >48 h. The attributable mortality among patients with ARDS and VA-LRTI was significantly higher than the mortality for VA-LRTI alone. Conversely, Forel et al. [42] prospectively analysed patients with ARDS and found that VAP occurred in almost one third of the patients. The authors also found an increased crude ICU mortality. However, this result was not significant after adjustment. Pseudomonas aeruginosa was followed by Methicillin-sensitive *Staphylococcus aureus* (MRSA) and other GNBs. Factors associated with mortality were the severity of disease and age. However, no particular aetiology was significantly associated with mortality. 

VAP research developments are commonplace in the literature. However, there is a growing field of study on respiratory infections in other areas, such VAT. These two entities are very close and, in many cases, VAP can be considered a continuum from VAT and previous colonization [43]. VA-LRTI as a whole might have some common ground with both VAT and VAP [3]. Martin-Loeches et al. [44] recently evaluated the clinical significance of VA-LRTI in patients with cardiovascular failure manifesting in a Sequential Organ Failure Assessment (mSOFA) cardiovascular score of 4 (at the time of the VA-LRTI diagnosis and present for at least 12 h). As expected, patients with cardiovascular failure had a higher mortality rate than those without it. However, the main focus in this prospective observational study was to determine the association of appropriate antibiotic treatments in patients with and without cardiovascular failure. In patients with cardiovascular failure, there was no association of appropriate antibiotic treatment and survival, while in those patients without cardiovascular failure, appropriate antibiotic treatment conferred a survival benefit only to patients with VAP. 

## 11. Conclusions

Lower respiratory tract infections, either from community or nosocomial acquisition, represent a significant burden for health care systems. The epidemiology in patients with CAP has changed in recent years with a higher incidence of GNBs. Some risk factors, such as age, are associated more often with GNB acquisition and the presence of anaerobes due to aspiration and dysphagia. Regarding infections of nosocomial origin, the old concept of early versus late onset episodes has been changed based on the different risk factors of the patients affected. Patients admitted to an ICU can develop VA-LRTI with two forms of severity: mild, in the form of tracheobronchitis, or severe, in the form of pneumonia. Both entities are often linked together. Appropriate antibiotic treatment plays a major role in VA-LRTI, with a different impact if the patients develop organ failure, such as septic shock or ARDS. Therefore, the absence of clinically useful biomarkers that can identify community acquired respiratory infections or those from a nosocomial origin and predict the severity of such infections on an individual basis remains a significant unmet need. 
